# Diagnostic Role of Extracellular Vesicles in Cancer: A Comprehensive Systematic Review and Meta-Analysis

**DOI:** 10.3389/fcell.2021.705791

**Published:** 2021-10-15

**Authors:** Shu-ya Liu, Yin Liao, Hossein Hosseinifard, Saber Imani, Qing-lian Wen

**Affiliations:** ^1^Department of Oncology, The Affiliated Hospital of Southwest Medical University, Luzhou, China; ^2^Department of Oncology, Chengdu Jinniu District People's Hospital, Chengdu, China; ^3^Department of Oncology, People's Hospital of Renshou, Meishan, China; ^4^Research Center for Evidence Based Medicine (RCEBM), Tabriz University of Medical Sciences, Tabriz, Iran

**Keywords:** extracellular vesicles, biomarker, cancer diagnostics, exosomal miRNA, meta-analysis

## Abstract

**Background:** Cancer-derived extracellular vesicles (EVs) are regarded to have significant function in most steps during cancer progression. This meta-analysis aims to investigate the accuracy of EVs as a biomarker in cancer diagnosis.

**Methods:** The diagnostic efficacy of EVs for different cancers was assessed using pooled sensitivity and specificity, diagnostic odds ratio (DOR), and overall area under the curve (AUC) of the summary receiver operating characteristic (SROC). The positive likelihood ratio (PLR) and negative likelihood ratio (NLR) were verified to estimate the diagnostic efficacy of EV at a clinical level.

**Results:** In all, 6,183 cancer patients and 2,437 healthy controls from 75 eligible studies reported in 42 publications were included in the study. The overall pooled sensitivity, specificity, PLR, NLR, and DOR were 0.62 (95% CI: 0.60–0.63), 0.76 (95% CI: 0.75–0.78), 3.07 (95% CI: 2.52–3.75), 0.34 (95% CI: 0.28–0.41), and 10.98 (95% CI: 7.53–16.00), respectively. Similarly, the AUC of the SROC was 0.88, indicating a high conservation of EVs as an early diagnostic marker. Furthermore, subgroup analysis suggested that the use of small EVs as a biomarker was more accurate in serum-based samples of nervous system cancer (*p* < 0.001). As a result, ultracentrifugation and quantification and size determination methods, such as Western blotting and ELISA were the most reliable identification methods for EV detection. We also indicated that increased secretion of EVs made them a capable biomarker for diagnosing cancer in elderly European individuals.

**Conclusions:** Our study provides evidence that EVs are a promising non-invasive biomarker for cancer diagnosis. Well-designed cohort studies should be conducted to warrant the clinical diagnostic value of EVs.

## Introduction

Cancer is still the second-leading cause of death worldwide, with an estimated 10 million cancer-related deaths reported in 2020 (Miller et al., 2019; Sung et al., 2021). According to the data reported by Globocan in 2020, lung cancer accounted for 18% of cancer-related deaths, followed by colorectal cancer (9.4%), liver cancer (8.3%), stomach cancer (7.7%), and breast cancer (6.9%) (Sung et al., 2021). Early screening for cancer in both healthy and high-risk populations can help detect cancer early, which greatly improves the opportunity for intervention and reduces mortality. Currently, most solid tumor diagnoses rely on imaging, and are then confirmed by tissue biopsy. However, the information obtained from a biopsy is influenced by the location from which the tumor tissue is taken and may not reflect the heterogeneity of the tumor. Moreover, because of the invasive nature of tissue biopsy, it cannot be performed routinely for monitoring prognosis. In addition, traditional tumor markers are also limited and often unsatisfactory for clinical use (Ju et al., 2014). Therefore, there is an urgent need for a novel non-invasive detection method that can fully clarify tumor characteristics and screen for early detection of cancer or accurately assess treatment efficacy (Ju et al., 2014; Marrugo-Ramírez et al., 2018). Compared with traditional tissue biopsy, liquid biopsy and blood-based biomarkers can detect tumor-related genetic changes and better identify disease recurrence or acquired resistance before clinical symptoms appear (Skotland et al., 2017; Wang et al., 2017; Marrugo-Ramírez et al., 2018).

As a circulating biomarker for cancer, extracellular vesicles (EVs) have gradually attracted wide attention in recent years (Petersen et al., 2020; Chen et al., 2021). EVs are cell-secreted nanoparticles involved in diverse pathological processes of tumors (He et al., 2018). Hypoxic, acidic, and inflammatory tumor microenvironments can enhance EV secretion (Parolini et al., 2009; Ramteke et al., 2015; Atretkhany et al., 2016). Generally, EVs reflect the genetic information of their original cancer cells, and can be isolated from various bodily fluids (Pisitkun et al., 2004; Michael et al., 2010). They have various constituents, including proteins, lipids, carbohydrates, DNA, and different RNA subtypes (Fujita et al., 2016). The abundance and composition of EVs often vary in different cancer types. Importantly, the miRNA in EVs obtained from blood samples and the protein profile of EVs show specificity and heterogeneity of tumors, suggesting that EVs may be promising biomarkers for various cancers (Conde-Vancells et al., 2008; Mitchell et al., 2008). Tumor-derived EVs deliver genetic information and further regulate recipient cells toward a pro-oncogenic phenotype (Salido-Guadarrama et al., 2014; Squadrito et al., 2014; Whiteside, 2016). Notably, growing evidence suggests that EVs are associated with the diagnosis of many malignant human cancers, including lung (Cazzoli et al., 2013), pancreatic (Madhavan et al., 2015), colorectal (Ogata-Kawata et al., 2014), prostate (Bryzgunova et al., 2016), and ovarian cancers (Meng et al., 2016). Although EVs have a strong potential for disease diagnosis and may aid therapeutic decision-making, further research efforts are required for viable clinical applications of EVs. In the field of clinical interpretation, detection of cancer-driving EVs in patients with no previous cancer history may dictate chemoprevention for cancer prevention or suppression, more frequent follow-ups for early cancer detection, as well as risk-reducing prophylactic surgeries such as appendectomy, colectomy, or mastectomy (Umwali et al., 2021; Xiao et al., 2021). Therefore, we conducted a quantitative systematic review along with a comprehensive meta-analysis based on published literature to confirm the diagnostic value of EVs in cancer patients in comparison with healthy subjects to suggest its potential as a non-invasive diagnostic tool for early cancer detection. Besides, we tried to document the evidence for using EVs as a diagnostic marker to predict other clinicopathological feature outcomes of cancer.

## Methods

### Search Strategy

**Figure 1:**
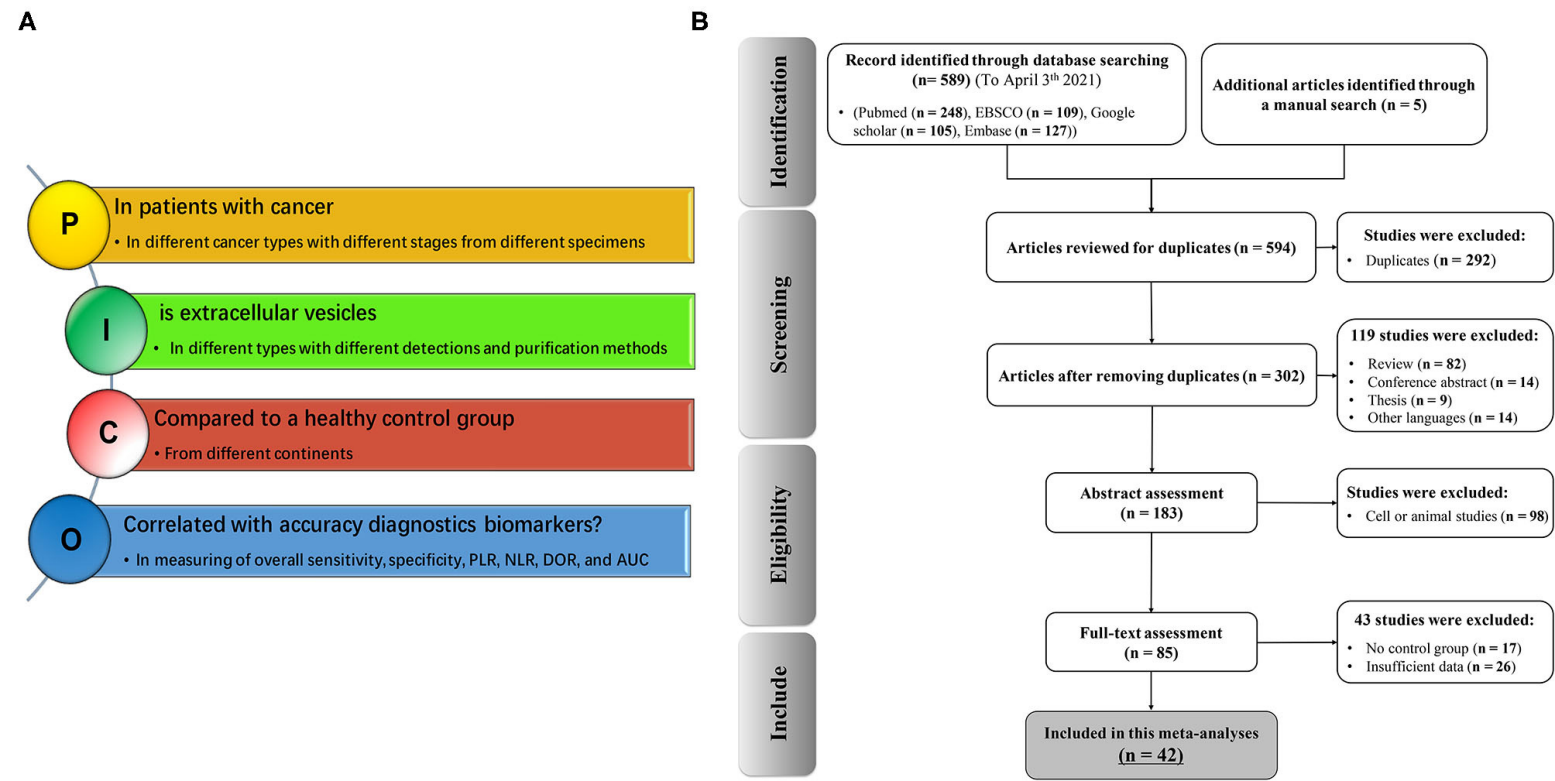
Flow chart of study selection according to the PICOs principles **(A)** and PRISMA **(B)** in the meta-analysis (*n* = number of studies).

We set up a comprehensive systematic search strategy and defined clinical issues according to the Population, Intervention, Comparison, Outcomes (PICOs) principle. The selection of clinical outcomes after using EVs as an early cancer biomarker is detailed in Figure 1A according to the PICOs principles. We conducted a comprehensive systematic literature search of studies published in English from inception to April 3, 2021 using Medline electronic databases (including PubMed, ISI Web of Science, Google Scholar, Vendor Information Page Database, and Embase) to identify all relevant studies. Our search included the following terms: “extracellular vesicles or EVs or EV or exosome or microvesicle,” “cancer or tumor or neoplasm or carcinoma,” and “prognosis or survival or outcome.” Alternative spellings and synonyms were combined by applying the Boolean “OR,” and main terms were linked using the Boolean “AND” to identify all relevant studies. The reference lists of all relevant articles were further reviewed for additional eligible publications.

### Study Inclusion/Exclusion Criteria

The current meta-analysis was conducted in accordance with the PICOs principle. Studies that met the following criteria were considered eligible: (i) cancer was confirmed by immunohistochemical or histochemical analysis; (ii) levels of EVs in tissue, plasma, serum, or other body fluids were measured; and (iii) relationship between EV secretion level and the diagnosis and prognosis of cancer, such as sensitivity and specificity, were reported in case-control study or could be measured from the provided data. The following studies were excluded: (i) non-English articles; (ii) reviews studies, conference abstracts, meetings, comments, letters, or experiments on cell line and animal models (iii); duplicate articles or continued studies of previous publications. (iv) studies with unqualified key data such as true positive (TP), false positive (FP), false negative (FN), true negative (TN), odds ratios (ORs) with 95% confidence intervals (CIs), and inadequate *P*-value; and (v) studies including cancer patients with additional systematic disorders and co-morbidities such as inflammations and/or autoimmune diseases.

### Data Extraction

Information from all eligible studies was separately recorded by two investigators (S. L. and Y. L.). The following key component data were collected from the included studies: first author's name, publication year, country origin, continent, cancer type, cancer stage, cancer category, specimen type, patient age group, therapy type, sampling size, EV purification and identification, detection methods, EV biomarkers, and true and false positives and negatives. Any inconsistency or disagreement in the research process was resolved through a debate and consultations. If a consensus could not be reached, a third investigator (S.I.) resolved these disagreements based on the original data. We also sent e-mails to the corresponding authors of qualified studies and asked for the original data, as well as any missing or additional information, required for our meta-analysis. Table 1 and Supplementary Table 1 show the summarized main demographics, clinicopathological information, and characteristics of EVs from all selected articles.

**Table 1:** Basic characteristics of the studies included in this meta-analysis.

**First author (Ref.)**	**Year**	**Country**	**Continent**	**Cancer**	Stage^*^	**Cancer category**	**Specimen**	**Age (median)**	**Therapy type**	**Sample size**		NOS^***^
										**Cases**	**Controls**	
Que et al. (2013)	2013	China	Asia	PDAC	I–IV	DSC	Serum	NR	NR	22	27	6
Cazzoli et al. (2013)	2013	Italy	Europe	NSCL	I	RSC	Plasma	70.5	NR	50	30	6
Wang et al. (2014)	2014	China	Asia	LSCC	I–III	RSC	Serum	59	SURG	52	49	7
Madhavan et al. (2015)	2014	Germany	Europe	PDAC	III–IV	DSC	Serum	64.8	NR	131	30	8
Ogata-Kawata et al. (2014)	2014	Spain	Europe	CRC	II–IV	DSC	Serum	NR	SURG	88	11	6
Matsumura et al. (2015)	2015	Japan	Asia	CRC	I–IV	DSC	Serum	65	SURG	209	16	7
Melo et al. (2015)	2015	Germany	Europe	BC	I–IV	ESC	Serum	NR	NR	32	100	7
Butz et al. (2016)	2015	Canada	America	RCC	NA	USC	Urine	NR	NR	109	51	6
Chiam et al. (2015)	2015	Australia	Europe	ESCC	III–IV	DSC	Serum	59.5	SURG + CT + RT	18	29	8
Zhou et al. (2017)	2016	China	Asia	NSCL	I–IV	RSC	Plasma	60	NR	141	124	5
Bryzgunova et al. (2016)	2016	Russian	Asia	PDAC	III	DSC	Urine	72	SURG	14	20	6
Samsonov et al. (2016)	2016	Russian	Asia	PC	I–III	USC	Urine	65	SURG + CT	35	35	6
Liu et al. (2016)	2016	USA	America	CRC	II–III	DSC	Serum	57	SURG	57	27	8
Zhang et al. (2018)	2016	China	Asia	RCC	I–III	USC	Serum	41	NR	82	80	8
Meng et al. (2016)	2016	Germany	Europe	EOC	I–IV	ESC	Serum	60	SURG + CT	163	20	9
Liu et al. (2017)	2016	China	Asia	NSCL	I–IV	RSC	Plasma	54.5	SURG + CT + RT	196	21	6
Machida et al. (2016)	2016	Japan	Asia	HCC	I–IV	DSC	Saliva	65	NR	12	13	6
Sandfeld-Paulsen et al. (2016)	2016	Denmark	Europe	NSCL	I–IV	RSC	Plasma	68.6	NR	107	54	7
Lea et al. (2017)	2017	USA	America	EOC	II–III	ESC	Plasma	NR	NR	34	10	8
Qu et al. (2017)	2017	China	Asia	HCC	I–IV	DSC	Serum	54.3	SURG	30	10	7
Lan et al. (2018)	2017	China	Asia	GBM	I–IV	NSC	Serum	46.2	SURG	60	43	8
Wang et al. (2017)	2017	China	Asia	ESCC	NA	DSC	Serum	60.8	NR	20	20	8
Skotland et al. (2017)	2017	Norway	Europe	PC	NA	ESC	Urine	NR	NR	15	13	6
Rodriguez et al. (2017)	2017	Norway	Europe	PDAC	I–III	DSC	Urine	67.2	CT^**^	28	19	5
Lai et al. (2017)	2017	USA	America	PDAC	NA	DSC	Plasma	NA	NA	3	6	7
Jin et al. (2017)	2017	China	Asia	NSCL	I	RSC	Plasma	61.3	NA	47	13	6
Yan et al. (2017)	2017	China	Asia	CRC	I–IV	DSC	Serum	58.6	CT	192	39	8
Shi et al. (2018)	2017	China	Asia	HCC	I–IV	DSC	Serum	65.7	SURG	126	21	7
Shiromizu et al. (2017)	2017	Japan	Asia	CRC	I	DSC	Serum	NR	NR	107	54	8
Arbelaiz et al. (2017)	2017	Spain	Europe	HCC	NA	DSC	Serum	64.3	SURG + CT	43	32	7
Tsukamoto et al. (2017)	2017	Japan	Asia	CRC	I–IV	DSC	Plasma	NA	NR	326	30	6
Xu et al. (2017)	2017	China	Asia	PC	I–IV	USC	Urine	69.45	NA	60	61	7
Yan et al. (2018a)	2018	China	Asia	CRC	I–IV	DSC	Serum	58.6	CT	168	20	6
Goto et al. (2018)	2018	Japan	Asia	PDAC	I–IV	DSC	Serum	64.1	SURG + CT + RT	32	22	7
Pan et al. (2018)	2018	Germany	Europe	EOC	I–IV	ESC	Plasma	60.3	SURG + CT	106	29	7
Wang et al. (2018)	2018	China	Asia	RCC	I–IV	USC	Serum	50.7	SURG	45	30	9
Yan et al. (2018b)	2018	China	Asia	CRC	I–IV	DSC	Serum	59.6	SURG + CT + RT	142	40	6
Kanaoka et al. (2018)	2018	Japan	Asia	NSCL	I–III	RSC	Plasma	72.5	SURG	285	24	7
Takahasi et al. (2018)	2018	Japan	Asia	PDAC	I–II	DSC	Plasma	NA	NR	50	20	7
Xu et al. (2019)	2019	China	Asia	NSCL	II–IV	RSC	Plasma	65.6	SURG + CT	43	20	7
Yu et al. (2019)	2019	China	Asia	GBM	I–IV	NSC	Serum	65.6	CT	12	32	8
Sakaue et al. (2019)	2019	Japan	Asia	PDAC	NA	DSC	Ascites	66.9	CT	19	49	8

*NSCLC, non-small-cell lung cancer; LSCC, laryngeal squamous cell carcinoma; PDAC, pancreatic ductal adenocarcinoma; PC, prostate cancer; CRC, colorectal cancer; BC, breast cancer; RCC, renal cell carcinoma; EOC, epithelial ovarian cancer; HCC, hepatocellular carcinoma; GBM, glioblastoma; ESCC, esophageal squamous cell carcinoma; RSC, respiratory system cancer; NSC, neuron system cancer; DSC, digestive system cancer; ESC, endocrine system cancer; USC, urinary system cancer; NR, not reported; NA, not available and/or not measurable; SURG, surgery; CT, chemotherapy; RT; Radiotherapy; NOS, the newcastle-ottawa scale*.

* *Malignant tumors classified according the tumor-node-metastasis (TNM) stage*.

** *Neoadjuvant chemotherapy and adjuvant therapy are categorized as chemotherapy*.

*** *The specific item information of NOS is available from http://www.ohri.ca/programs/clinical_epidemiology/oxford.asp*.

### Quality Assessment

The systematic review and meta-analysis were conducted according to the recommendations of the Preferred Reporting Items for Systematic Reviews and Meta-Analyses (PRISMA) guidelines (46). In case of controversial judgement, the diagnostic accuracy of each study was evaluated independently by three authors using the Quality Assessment of Diagnostic Accuracy Studies 2 (QUADAS-2) tool, which consists of four domains: patient selection, index text, reference standard, and flow timing (47). Any discrepancies were resolved through a discussion. Each assessment was subjected to seven questions that were answered with “yes,” “no,” or “unclear.” The answer of “yes” indicated that a study's risk of bias was low, whereas “no” and “unclear” indicated a high risk of bias.

### Statistical Analysis

The systematic meta-analysis was conducted by using the R software version 4.0.1 (R Foundation for Statistical Computing, Vienna, Austria) and package “mada” version 0.5.9. Pooled sensitivity, pooled specificity, negative likelihood ratio (NLR), positive likelihood ratio (PLR), and diagnostic odds ratio (DOR) were calculated with corresponding 95% CIs to evaluate the diagnostic value of EVs. Heterogeneity of studies in meta-analysis refers to the variation in study outcomes between studies (Gavaghan et al., 2000). In order to interpret our results, we used the Stats Direct calls statistics model for measuring heterogeneity in meta-analysis “non-combinability” statistics (Higgins et al., 2003). Correspondingly, the heterogeneity of the combined DOR was assessed via Cochran's *Q* test and Higgins *I*^2^ statistic from non-threshold effect (Hedges and Pigott, 2001). Subgroup analysis was conducted to determine the source of the existing heterogeneity between EVs and the available sub-analysis parameters such as ethnicity, cancer type, specimen type, age group, therapy type, EV type, EV purification and identification methods, and EV secretion level. *p* < *0.05* and/or *I*^2^ > *50%* indicated statistical heterogeneity. Data are reported as mean ± standard deviation (SD) or median (range), including a description of qualitative variables such as numbers and percentages. The diagnosis accuracy of included studies was presented on the summary receiver operating characteristic (SROC) curve. Subgroup analysis was conducted to determine the source of heterogeneity. Publication bias was evaluated by a funnel plot and Egger's regression test. A value of “Pr > |z|” <0.05 was considered to indicate potential publication bias. All the reported *p* values were two-sided, and a *p* < 0.05 was considered to indicate statistical significance.

## Results

### Selection of Studies

A schematic PRISMA flowchart describing the screening and inclusion and exclusion criteria is shown in Figure 1B. The literature search yielded 594 potentially relevant publications, which were related to the topic of cancer biomarkers and included EVs. Of these, 302 studies eligible for inclusion were confirmed after excluding duplicate studies (292 studies). Subsequently, 119 studies with unrelated content were excluded. The remaining full-text articles of 183 studies were assessed for suitability. However, 98 studies were dismissed because of apparent irrelevance, and 43 studies were removed because of insufficient data. Finally, 42 articles with 75 studies were included in this meta-analysis. In detail, eight articles (Que et al., 2013; Wang et al., 2014; Chiam et al., 2015; Melo et al., 2015; Bryzgunova et al., 2016; Machida et al., 2016; Zhang et al., 2018; Yu et al., 2019) analyzing of two different EVs, four articles (Meng et al., 2016; Samsonov et al., 2016; Liu et al., 2017; Goto et al., 2018) analyzing of three EVs, one research article (Pan et al., 2018) with analyzing of four different EVs, two articles (Arbelaiz et al., 2017; Shiromizu et al., 2017) analyzing five different EVs, and one article with evolution of seven different EVs (Ogata-Kawata et al., 2014) were studied to determine the diagnostic accuracy of EVs in cancer patients (Supplementary Table 1). The detailed process is shown in Figure 1.

### Study Characteristics and Quality Assessment

**Figure 2:**
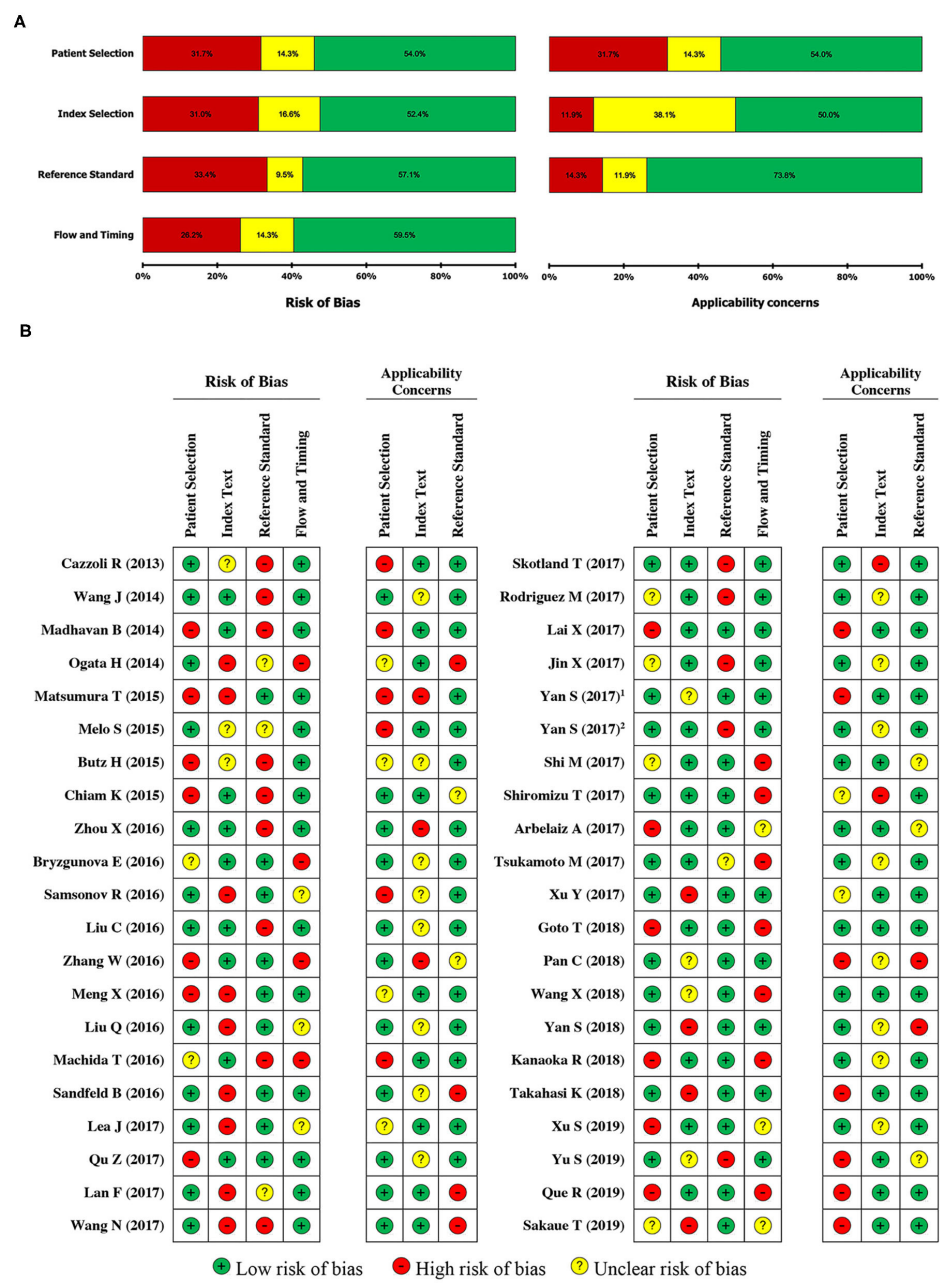
Risk of bias graph. The overall risk of bias was regarded as low in all qualified studies as per the QUADAS-2 assessment. The reviewers' decisions about each risk of bias and applicability concerns graph **(A)** presented as percentages across of single selected studies **(B)**. “+” low risk of bias; “–” high risk of bias; “?” unclear risk of bias.

The main characteristics of all included literature are presented in Table 1 in the order of year of publication. In total, 8,620 subjects (6,183 cancer patients and 2,437 healthy controls) from studies reported between 2013 and 2019 were included in this meta-analysis, and histologically classified into five types of cancers: digestive system cancer (*n* = 22), respiratory system cancer (*n* = 8), endocrine system cancer (*n* = 5), urinary system cancer (*n* = 5), and nervous system cancer (*n* = 2). We did not find any study between 2019 and 2021 according to the inclusion/exclusion criteria and PICOs principle. The studies were conducted mostly in Asia (27 studies, 64%) and Europe (11 studies, 26%), and four studies were conducted in America (10%), with no study conducted in the African continent. Among these studies, EV secretion levels were measured in serum samples (*n* = 22), plasma samples (*n* = 12), urine samples (*n* = 6), saliva sample (*n* = 1), and ascites sample (*n* = 1). The major characteristics of EVs in the included studies are shown in Supplementary Table 1. All 42 studies were methodologically assessed based on the Newcastle-Ottawa scale (NOS) and QUADAS-2 protocols. In general, the average NOS score was about 7 out of 12, which could almost be classified as the high-quality group. Table 1 showed the NOS score of each study. Furthermore, QUADAS-2 results suggested that significant bias did not exist in the current meta-analyses (Figure 2).

### Outcome of the Meta-Analysis

The main results of this meta-analysis with regard to the relation between EV secretion and cancer risk are shown in Table 2. A random effect model was used to calculate statistically significant combined OR and 95% CIs (Table 2). Accordingly, we tried to explain the sources of heterogeneity from random sample sources to accurately determine the significance of EVs. The threshold effect of spearman correlation coefficient was the main reason of heterogeneity in the test accuracy studies (Zamora et al., 2006). To clarify the source of heterogeneity, we further performed subgroup analyses.

**Table 2:** Meta-analysis results for the association between extracellular vesicles and cancer risk.

	**No. of studies**	**Sample size (cases/controls)**	***x*** **^2^**	***I*** **^2^ (%)**	**Pooling model**	**Pooled**	**OR (95% CI)**	***P*** **-value**
Sensitivity	75	6183/2437	1689.52	95.60	R	0.62	0.60-0.63	<0.001
Specificity	75	6183/2437	380.38	80.50	R	0.76	0.75-0.78	<0.001
PLR	75	6183/2437	429.81	82.80	R	3.07	2.52-3.75	<0.001
NLR	75	6183/2437	1572.96	95.30	R	0.34	0.28-0.41	<0.001
DOR	75	6183/2437	514.13	85.60	R	10.98	7.53-16.00	<0.001

*OR, odds ratio; CI, confidence interval; PLR, positive likelihood ratio; NLR, negative likelihood ratio; DOR, diagnostic odds ratio; x^2^, chi-squared; R, randomize model*.

### Diagnostic Accuracy

**Figure 3:**
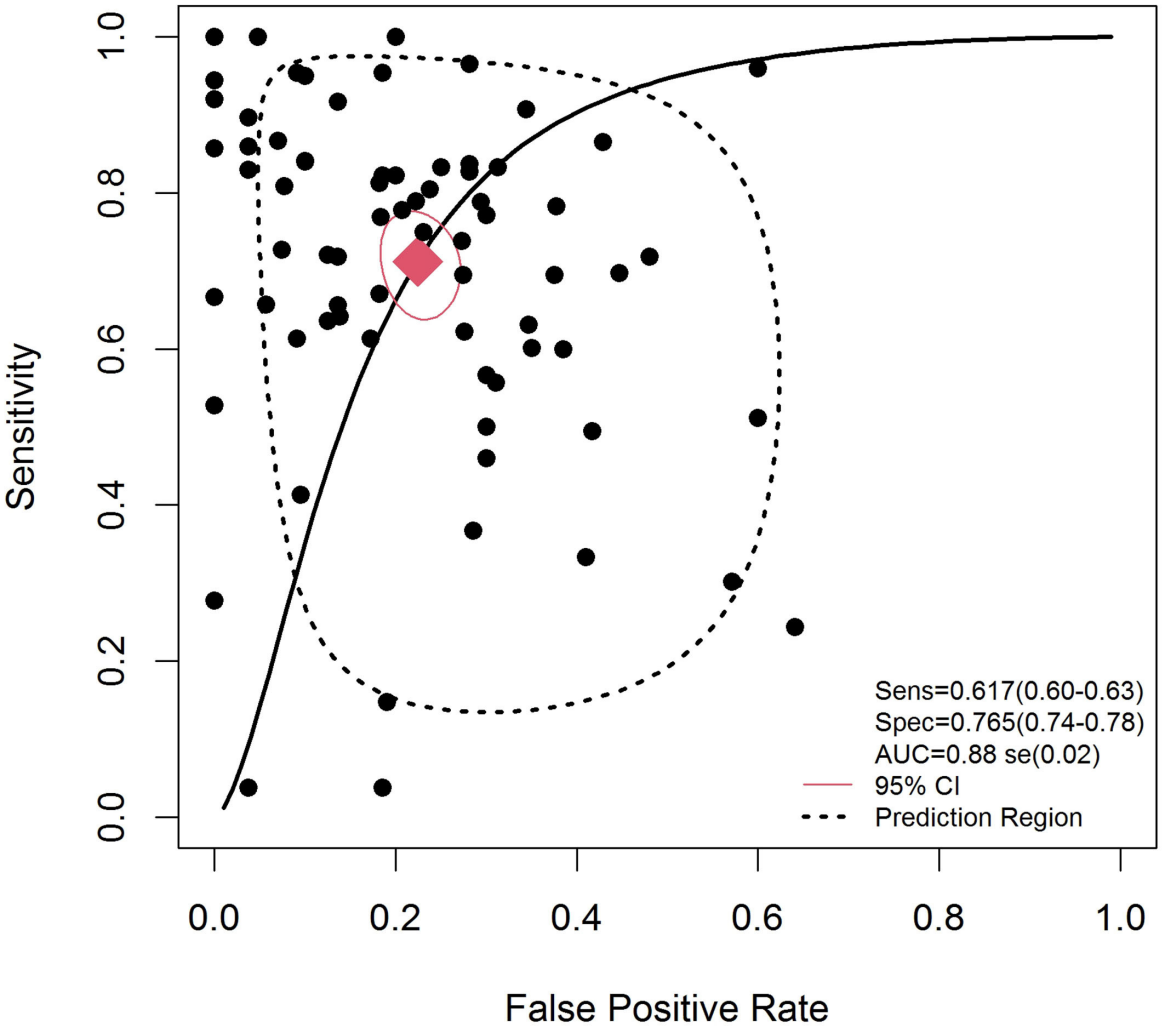
Summary receiver operating characteristic curve for EVs in the diagnosis of cancer. Weights are from random effect analysis.

The effect of heterogeneity on the diagnostic threshold was evaluated based on the Spearman correlation coefficient. Figure 3 demonstrates the forest plots of pooled sensitivity, specificity, and DOR with their 95% CIs for individual studies. Based on the results, the overall pooled results for sensitivity, specificity, PLR, NLR, and DOR with their 95% CIs were 0.62 (95% CI: 0.60–0.63), 0.76 (95% CI: 0.75–0.78), 3.07 (95% CI: 2.52–3.75), 0.34 (95% CI: 0.28–0.41), and 10.98 (95% CI: 7.53–16.00), respectively, showing that there was no heterogeneity from the threshold effect of sensitivity and specificity (*p* = *0.004*). The SROC curve for the included studies is indicated in Figure 3 with an overall AUC of 0.88.

### Subgroup Analysis

**Figure 4:**
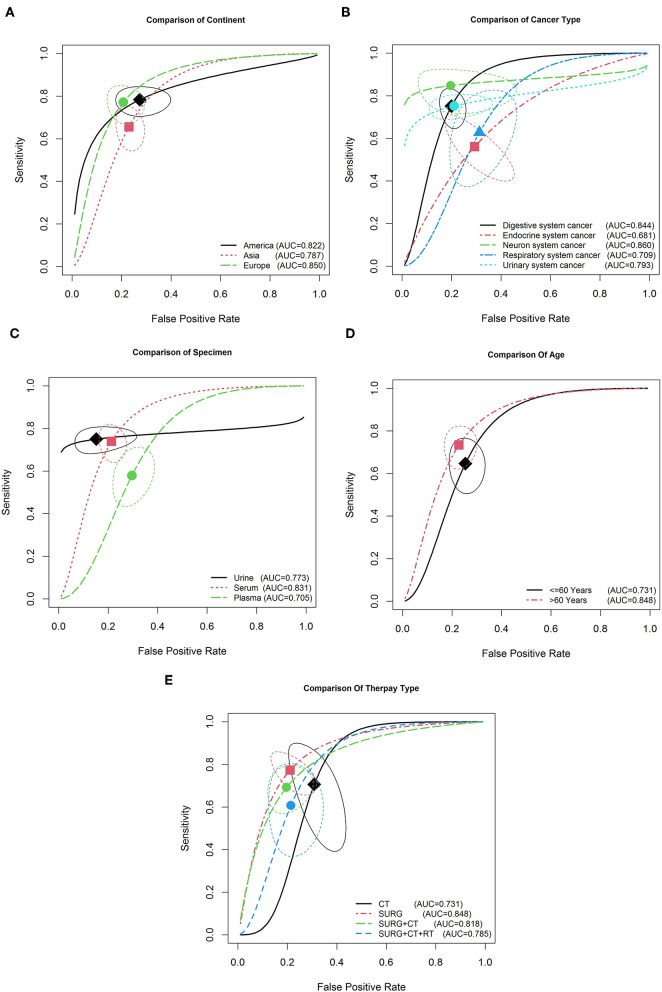
Summary receiver operating characteristic curve for EVs and subgroup analysis based on continents **(A)**, cancer type **(B)**, specimen type **(C)**, patient age **(D)**, and therapy type **(E)**.

Subgroup analysis was conducted to determine the source of heterogeneity between the EVs and available sub-analysis parameters. Table 3 reveals that none of the above mentioned covariates contributed to heterogeneity (all *p* > *0.05*). Therefore, based on these covariates, the pooled sensitivity, specificity, and AUC for important sub-analysis parameters were measured. The results showed higher accuracy in American than in Asian and European populations, with a sensitivity of 0.79 (95% CI: 0.72–0.84), specificity of 0.74 (95% CI: 0.64–0.83), and AUC of 0.822 (Figure 4A). As shown in Figure 4B and Table 3, EVs are a potentially accurate diagnostic biomarker in nervous system cancers (*P* = 0.24, 95% CI: 0.15–0.35) compared with the other four types, with a sensitivity of 0.85 and specificity of 0.80. With regard to specimen type, EVs showed a higher diagnostic accuracy for cancer detection in urine samples (with an 0.79 sensitivity and 0.82 specificity) than in plasma (with an 0.50 sensitivity and 0.72 specificity) and serum (with an 0.66 sensitivity and 0.77 specificity) samples, with an AUC of 0.78 (Table 3 and Figure 4C). Furthermore, subgroups analysis was performed based on age (≤60 vs. >60). The sensitivity and specificity of the population aged over 60 years (33 studies with 2,013 cases) were 0.65 (95% CI: 0.63–0.68) and 0.77 (95% CI: 0.74–0.8) respectively, whereas those of the population aged <60 years (20 studies with 2,216 cases) were 0.58 (95% CI: 0.56–0.6) and 0.73 (95% CI: 0.7–0.76), respectively. As a result, a higher diagnostic accuracy for cancer detection was observed in individuals aged over 60 years than in those aged <60 years, with AUCs of 0.848 vs. 0.731 (Figure 4D). Furthermore, subgroup analysis of different therapy types showed a higher accuracy of EVs in patients undergoing surgery (Figure 4E). Interestingly, our results show that EVs isolated from patients undergoing surgery show a higher accuracy than those isolated from patients undergoing chemotherapy or radiotherapy (Figure 4E). As seen in Table 3, the pooled sensitivity and specificity of EVs isolated from patients undergoing only surgery (18 studies with 1,560 cases) were 0.68 (95% CI: 0.66–0.71) and 0.79 (95% CI: 0.75–0.83), respectively. However, EVs isolated from patients undergoing surgery and chemotherapy (16 studies with 1,248 cases) showed a close diagnostic accuracy to that of EVs isolated from patients undergoing only surgery (with a sensitivity of 0.71 and specificity of 0.77).

**Table 3:** Subgroup analysis of the included studies.

**Subgroup analyses**		**No**.	**Case/control**	**Sensitivity (95% CI)**	**Specificity (95% CI)**	**PLR (95% CI)**	**NLR (95% CI)**	**DOR (95% CI)**
Ethnicity	America	4	203/94	0.79 (0.72–0.84)	0.74 (0.64–0.83)	2.92 (2.11–4.20)	0.29 (0.21–0.38)	10.32 (5.92–18.01)
	Asia	41	3685/1493	0.55 (0.53–0.56)	0.77 (0.74–0.79)	2.70 (2.09–3.50)	0.40 (0.30–0.52)	7.64 (4.70–12.41)
	Europe	30	2295/850	0.70 (0.68–0.72)	0.76 (0.73–0.79)	3.92 (2.72–5.66)	0.28 (0.21–0.39)	20.24 (10.10–40.57)
Cancer type	DSC	44	3366/1252	0.65 (0.64–0.67)	0.79 (0.77–0.81)	3.62 (2.68–4.90)	0.27 (0.19–0.38)	15.69 (8.99–27.41)
	ESC	10	994/299	0.55 (0.52–0.58)	0.70 (0.64–0.76)	2.66 (1.24–5.73)	0.54 (0.39–0.74)	5.57 (1.70–18.25)
	NSC	3	84/107	0.85 (0.76–0.92)	0.80 (0.71–0.87)	4.40 (1.67–11.63)	0.17 (0.01–0.28)	26.31 (6.92–99.99)
	RSC	10	1258/372	0.49 (0.46–0.51)	0.69 (0.64–0.74)	1.79 (1.26–2.54)	0.52 (0.36–0.75)	4.04 (1.88–8.70)
	USC	8	483/407	0.76 (0.72–0.80)	0.76 (0.71–0.80)	3.40 (2.22–5.20)	0.32 (0.25–0.39)	12.36 (6.49–23.57)
Specimen	Urine	9	345/289	0.79 (0.74–0.83)	0.82 (0.77–0.86)	5.50 (2.78–10.90)	0.29 (0.20–0.42)	26.19 (9.26–74.10)
	Serum	47	3804/1617	0.66 (0.64–0.67)	0.77 (0.74–0.79)	3.43 (2.60–4.54)	0.29 (0.22–0.38)	14.22 (8.30–24.37)
	Plasma	16	1991/456	0.50 (0.47–0.52)	0.72 (0.67–0.76)	1.87 (1.42–2.45)	0.58 (0.47–0.72)	3.64 (2.21–5.93)
	Other	3	43/75	0.67 (0.52–0.81)	0.73 (0.62–0.83)	2.83 (1.09–7.35)	0.44 (0.28–0.68)	7.24 (1.84–28.49)
Age	< = 60	20	2216/772	0.58 (0.56–0.6)	0.73 (0.7–0.76)	2.52 (1.91–3.32)	0.42 (0.32–0.53)	7.05 (4.15–11.97)
	>60	33	2013/934	0.65 (0.63–0.68)	0.77 (0.74–0.8)	3.16 (2.32–4.31)	0.3 (0.21–0.42)	13.48 (7.27–25)
Therapy	SURG	18	1560/386	0.68 (0.66–0.71)	0.79 (0.75–0.83)	3.42 (2.39–4.91)	0.26 (0.17–0.41)	15.52 (7.43–32.42)
	SURG + CT^*^	16	1248/461	0.71 (0.68–0.73)	0.77 (0.73–0.8)	3.49 (2.29–5.3)	0.33 (0.24–0.44)	14.39 (6.86–30.17)
	SURG + CT + RT	9	862/227	0.44 (0.41–0.47)	0.81 (0.75–0.86)	2.77 (1.68–4.58)	0.48 (0.34–0.69)	6.3 (2.62–15.13)
Type of EVs^**^	lEVs	41	5064/1681	0.57(0.56–0.59)	0.76(0.73–0.78)	2.67(2.10–3.34)	0.39(0.31–0.51)	8.42(5.34–13.28)
	sEVs	34	1119/756	0.67(0.65–0.69)	0.81(0.75–0.85)	3.71(2.62–5.25)	0.29(0.22–0.40)	14.61(7.71–27.68)
EVs purification	Isolation kit^***^	26	2074/875	0.56(0.53–0.58)	0.73(0.7–0.76)	2.52(1.9–3.34)	0.4(0.31–0.53)	7.41(4.31–12.74)
	UC^#^	49	4109/1562	0.65 (0.63–0.66)	0.78(0.76–0.8)	3.51(2.66–4.64)	0.31(0.24–0.41)	13.92(8.3–23.34)
EVs identification	NA	11	703/312	0.56 (0.52–0.6)	0.78(0.73–0.83)	2.59(1.44–4.68)	0.36(0.14–0.89)	8.13(2.64–25.06)
	Microscopic^##^	31	2579/1075	0.54 (0.52–0.56)	0.72(0.69–0.75)	2.61(1.98–3.43)	0.41(0.32–0.53)	8.2(4.61–14.6)
	Exosomal surface biomarkers^###^	20	1817/378	0.68(0.66–0.7)	0.8(0.77–0.83)	3.6(2.09–6.18)	0.26(0.13–0.51)	14.86(8.36–26.41)
	Quantification and size determination^&^	13	1084/672	0.74 (0.71–0.76)	0.82(0.77–0.85)	4.35(2.85–6.62)	0.31(0.24–0.41)	16.37(5.91–45.3)
EVs secretion level	Increase	62	5123/2095	0.63 (0.61–0.64)	0.77(0.76–0.79)	3.22(2.61–3.98)	0.32(0.26–0.4)	12.31(8.19–18.5)
	Decrease	11	1032/302	0.56 (0.53–0.59)	0.69(0.63–0.74)	1.99(1.2–3.3)	0.58(0.41–0.81)	4.21(1.73–10.23)

*EVs, extracellular vesicles; DSC, digestive system cancer; ESC, endocrine system cancer; NSC, neuron system cancer; RSC, respiratory system cancer; USC, urinary system cancer; SURG, surgery; CT, chemotherapy; RT; Radiotherapy; lEVs, large EVs; sEVs, small EVS; UC, ultracentrifuge*.

* *Neoadjuvant chemotherapy and adjuvant therapy are categorized as chemotherapy*.

** *EVs sedimenting at 100,000 × g into is categorized as a small EVs (sEVs) and EVs sedimenting at 2,000 × g is categorized as a large EVs (lEVs, large fragments of cells, large apoptotic bodies) (Mateescu et al., 2017; Slomka et al., 2018)*.

*** *Isolation kit refer to the standard total extracellular vesicles isolation kit media and urine for biomarker analysis*.

# *Using the differential ultracentrifugation range (>100,000 ×g (100,000–200,000 ×g) for 2 h) for isolation of different exosome isolation methods yield different amount of exosomes (Patel et al., 2019)*.

## *Microscopic methods for EVS identification is contended the transmission electron microscope, atomic force microscopy and selected reaction monitoring methods*.

### *CD81, CD63, CD64, CD65, and CD66 are a cell surface glycoprotein exosomal cell surface markers that is using mostly for isolation isolating exosomes from tissue culture media and urine for biomarker analysis (Konoshenko et al., 2018)*.

& *Quantificative methods for EVS identification is the quantificative and size determination based on the western blot and ELISA*.

**Figure 5:**
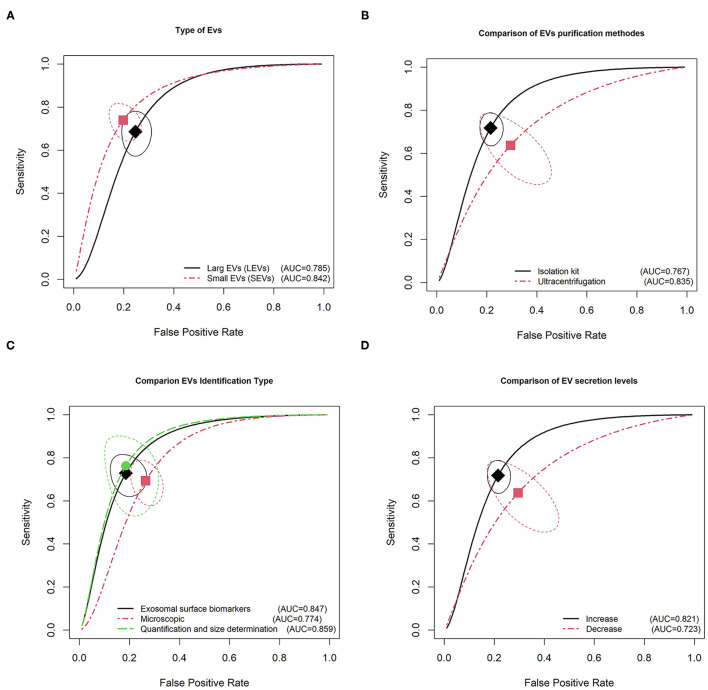
Summary receiver operating characteristic curve for EVs and subgroup analysis based on type of EVs **(A)**, purification methods **(B)**, identification type **(C)**, and secretion level **(D)**.

Figure 5 shows the results of the SROC analysis according to the characterization parameters of EVs, such as type of EVs, purification methods, identification methods of EVs, and EV secretion level. We categorized EVs sedimenting at 100,000 × *g* into small EVs (sEVs), those sedimenting at speeds lower than 20,000 × *g* into medium EVs (mEVs, microvesicles, ectosomes), and those sedimenting at 2,000 × *g* into large EVs (lEVs, large fragments of cells, large apoptotic bodies) (Mateescu et al., 2017; Slomka et al., 2018). As shown in Table 3, 5,064/1,681 cases/controls in 41 studies were compared with 1,119/756 cases/controls in 34 studies with sEVs. The sEVs showed higher pooled sensitivities and specificities than lEVs (0.67 vs. 0.57 and 0.81 vs. 0.76, respectively; Figure 5A). Meanwhile, with regard to purification methods, the ultracentrifugation method (range 100,000–200,000 × *g* for 2 h) showed higher sensitivity and specificity than kit-based isolation methods (0.65 vs. 0.56 and 0.78 vs. 0.73, respectively). The kit-based isolation methods depend on the optimization step for rapidly isolating EVs from the tissue culture media (Figure 5B). In this study, we categorized EV identification methods in three main groups, microscopic methods, identification based on exosomal surface biomarkers, and quantification and size determination methods. Microscopic methods for EV identification use the transmission electron microscope, atomic force microscopy, and selected reaction monitoring methods. Our results show that CD81, CD63, CD64, CD65, and CD66 are cell surface glycoproteins; exosomal cell surface markers were the surface biomarkers mostly used for the identification of exosomes from tissue culture media and urine (Konoshenko et al., 2018). Also, Western blotting and ELISA were the most reported quantification methods for EV identification (Table 3). Our meta-analysis results show that the quantification and size determination methods such as Western blotting and ELISA were the most reliable identification methods with a sensitivity of 0.74 (CIs: 0.71–0.76), specificity of 0.82 (CIs: 0.77–0.85), and AUC of 0.86 (Figure 5C). However, identification based on the EV surface biomarkers has close diagnostic accuracy to EVs identification (with a sensitivity of 0.68 and specificity of 0.80). In univariate logistic regression analysis, increased secretion of EVs showed higher diagnostic accuracy for cancer detection compared with decreased secretion of EVs, with the sensitivities of 0.63 vs. 0.56, specificities of 0.77 vs. 0.69, and AUCs of 0.821 vs. 0.723 (Figure 5D). Therefore, increased secretion of EVs could be a promising biomarker for cancer diagnosis.

### Publication Bias and Sensitivity Analysis

**Figure 6:**
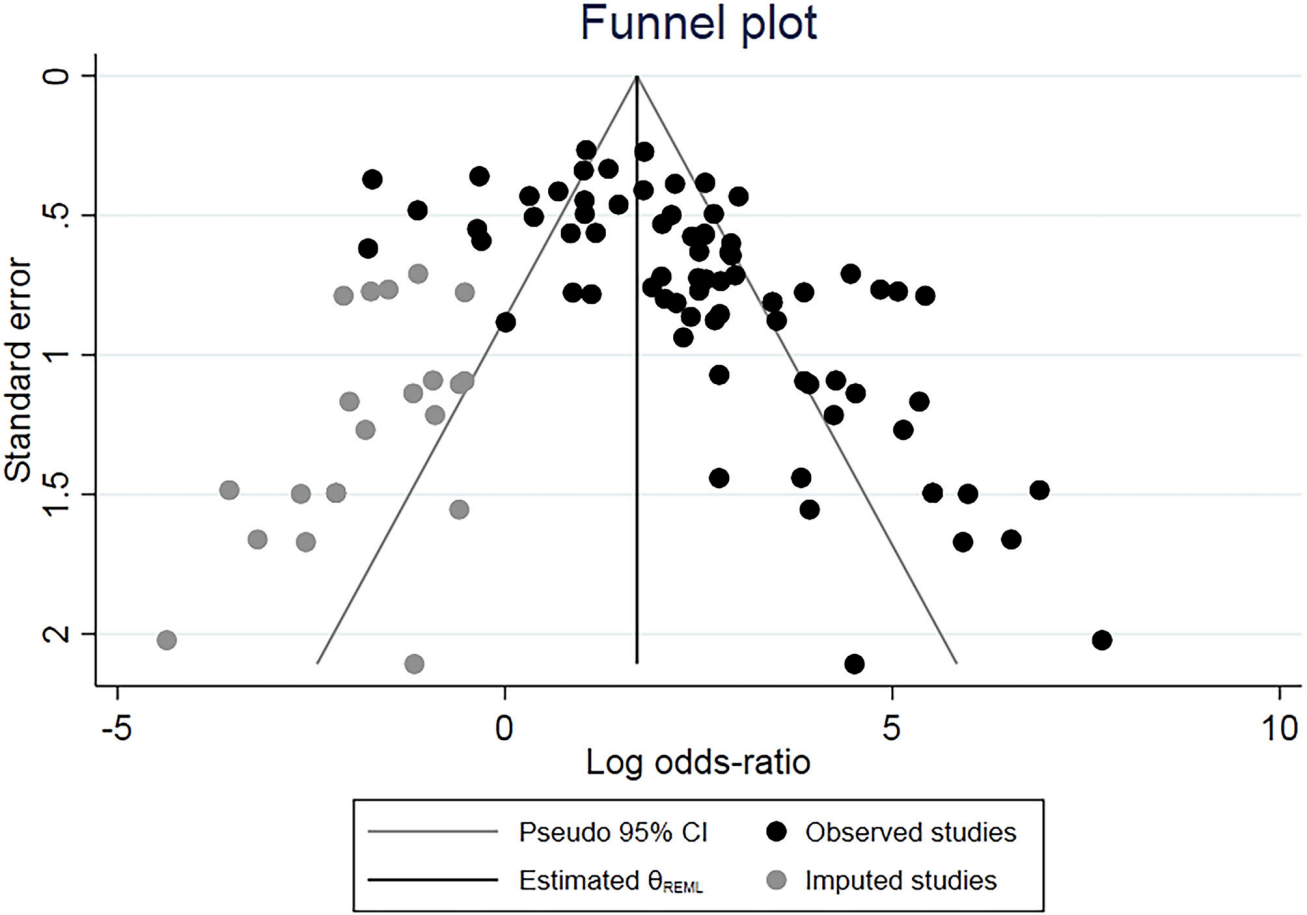
Contour-enhanced funnel plots for the detection of publication bias. Each point represents one of the 74 studies for the specified association. The size of each circle is proportional to the percentage weight that each study contributes to the HRs. These plots indicate that some studies were in significant areas where *p* ≤ *0.01* (solid lines). Solid triangles refer to included studies and X's refer to filled studies. The vertical axis represents the standard error of logarithmic HR limits.

Funnel plots and Egger's test were performed to estimate publication bias. These were conducted by precluding a single study at a time and significant differences between events and hypothesis were observed (Figure 6) (Tobias, 1999). The trim-and-fill method was employed and generally resulted in a confidence interval of 0.09 (95% CI: 0.04–0.14) using the unadjusted random-effect model. Additionally, this test identified 20 articles of all included studies with possible publication bias, but no significant effect on our main findings was found (*P* = 0.1038) and the high level of heterogeneity (*I*^2^ = 69%) remained (Figure 6). The resulting shape of the funnel plot and Egger's test provided no statistical evidence for publication bias (*t* = *1.16* and *p* = *0.164*). Hence, no noticeable evidence for significant publication bias in our meta-analysis was observed, which indicates that our meta-analysis results are stable and credible.

## Discussion

It is well-established that cancer cell-derived EVs released into a tumor microenvironment and circulation can promote tumor progression and metastasis by inducing matrix remodeling, angiogenesis, inflammation, and metastatic niche formation (Dong et al., 2002; Hoshino et al., 2015; Guo et al., 2019). Mechanically, cancer cell-derived exosomes promote tumor growth by triggering changes in naïve mesenchymal stem cells (MSCs) to form pro-inflammatory MSC in many solid tumors such as melanoma (Peinado et al., 2012), ovarian cancer (Feng et al., 2019), prostate cancer (Soekmadji et al., 2017), and breast cancers (King et al., 2012). Similarly, exosomal miRNA and lncRNA premetastatic niches develop in many cancers and modulate cell proliferation and motility/invasion (Soekmadji et al., 2017). Similarly, tumor-derived EVs, mostly exosomal miRNA and lncRNA, provide a genetic landscape, which promotes endothelial cell angiogenesis, and creates conditions for tumor growth, making this molecule a promising candidate as a biomarker to reflect various physiological and pathological states of cancer cells (Skog et al., 2008; Keller et al., 2011). This evidence indicates that EV-associated miRNAs are not only promising diagnostic and prognostic biomarkers but also important therapeutic targets as their secretion can reflect the information isolated from millions of oncogenes during tumor aggressiveness, resistance to chemotherapy, and tumor immune-escape. Several studies have identified candidate tumor-derived EV biomarkers that can be considered as cost-effective and non-invasive prospective biomarkers for cancer patients because they can be detected in bio fluid samples of cancer patients (D'Souza-Schorey and Clancy, 2012; Feng et al., 2019). These results propose that tumor-derived EV biomarkers can be used in a clinical setting if they are specific to a particular cancer type, which was partially demonstrated in our study.

With these assumptions and foreground, in this comprehensive study, we collected all available articles and performed a meta-analysis to confirm the diagnostic value of different types of EVs in different cancers. We aimed to understand the relationship of EVs as a diagnostic marker to predict other clinicopathological features and outcomes of different cancers, such as cancer type, specimen type, sample size, and cancer grade. As expected, the pooled HR from our results showed that the EV secretion levels have potential value for the diagnosis of cancer. To the best of our best knowledge, no meta-analysis has investigated the diagnostic value of EVs in cancers by displaying consistent, statistically significant changes in EV secretion level. In our meta-analysis of 42 articles with 75 studies, which included different types of cancers, the pooled sensitivity and specificity were 0.617 and 0.765, respectively; these are statistical measures that help determine the diagnostic value of EVs in cancer. In this comprehensive study, the assessment of diagnostic accuracy of EVs at a clinical level was verified by PLR and NLR tests. The values of PLR and NLR were 3.07 and 0.34, respectively, which demonstrated that the probability of a TP diagnosis is 3.07 times higher than the probability of a FP diagnosis and that there is a 34% error rate in the individuals testing negative. Specifically, the upper left corner SROC curve is the perfect test to evaluate the diagnostic value (Walter, 2002); our overall AUC of SROC curve was 0.88, which is considered to be in a good range of SROC curve statistically (the good range of AUC: 0.75–0.92) (Moher et al., 2009). This indicates that EVs are highly accurate as a biomarker for detecting cancer. Our results clearly illustrated that exosomal miRNA had a better accuracy with regard to cancer detection than exosomal protein, with AUCs of 0.811 vs. 0.810.

Recently, many studies have demonstrated that EV-packaged mRNAs are the most reported and well-evaluated extracellular RNAs (exRNAs) enriched in EVs that can serve as biomarkers for cancer (Cazzoli et al., 2013; Que et al., 2013; Ogata-Kawata et al., 2014; Wang et al., 2014; Chiam et al., 2015; Madhavan et al., 2015; Matsumura et al., 2015; Melo et al., 2015; Butz et al., 2016). Numerous pro-angiogenic miRNAs present in EVs released from breast cancer (miR-100, miR-222, miR-30a and miR-17), lung cancer (miR-146a, miR-100-5p), and ovarian cancer (miR-21) samples may contribute to new blood vessel formation and have been proposed as a reliable tumor biomarker (Que et al., 2013; Ogata-Kawata et al., 2014; Wang et al., 2017; Pan et al., 2018; Zhang et al., 2018). EV abundance and composition are also altered in individuals with cancer (Hu et al., 2021). As a main type of EVs, sEVs can provide a protective, enriched source of miRNA and increase the stability of endogenous miRNA (Hu et al., 2021). Goto et al. demonstrated that exosomal miRNA191, miRNA21, and miRNA451a are superior to serum circulating miRNAs in establishing a diagnosis of pancreatic neoplasms (Goto et al., 2018). Similarly, there is a possibility that the distinct protein or lipid profiling of sEVs was performed for identifying molecular features with novel biomarker potential diagnostic values, specifically in urine or blood samples of cancer patients (Momen-Heravi, 2017). However, little is known about clinical applicability of using protein or lipid profiling composition of EVs for detecting different stages of cancer.

Other results with different subgroups were relatively consistent with our main findings, indicating that our findings are reliable. Our systematic search clearly indicated that serum-based isolated EVs (AUC: 0.831) are more accurate diagnostic biomarkers than plasma or urine-based EVs. Recently, several studies have reported that the biological fluid source of exRNA is associated with the histological grade of cancer. Surely, most clinical studies have evaluated EVs as cancer biomarkers in whole blood, urine, and cerebrospinal fluids rather than in tissue. For example, serum miR-34a levels were associated with the last stage of breast cancer and serum-based tumor markers were the most effective screening tool for the detection of metastatic breast cancer (Taplin et al., 2008; Imani et al., 2017). Moreover, the secretion level of serum EVs of miR-18a, miR-221, and miR-224 has been suggested to be a potential diagnostic biomarker in hepatocellular carcinoma (Sohn et al., 2015; Lai et al., 2017; Rodriguez et al., 2017). However, no significant association between serum-based EVs and clinicopathological features of tumors, such as hormone receptors and lymph node metastasis, has been observed (Erbes et al., 2015; Mishra et al., 2015).

Owing to the existence of significant heterogeneity, a meta-analysis and subgroup analysis were performed to identify other related factors affecting the heterogeneity. We observed that different isolation methods of EVs even within the same approach for EV isolation interfered with verification and yielded different amounts of EVs (Patel et al., 2019). As expected, our results confirmed that ultracentrifugation techniques of EVs had a higher accuracy for diagnosing cancers. Ultracentrifuges are a classic technique considered as a gold standard for EV collation (Stam et al., 2021). These methods allow for the purification of subpopulations of EVs such as exosomes according to size differentiation (Sunkara et al., 2019; Stam et al., 2021). Also, differential ultracentrifugation is the most available method for EV isolation that has been integrated with mass spectrometry techniques with unique peptides in a multicenter study (Yan et al., 2009). However, the excellence of proper isolation of EVs using ultracentrifugation techniques depends on the materials available in the laboratory, type of EVs, samples, type of cancer, and the required amount of EVs (Momen-Heravi, 2017; Stam et al., 2021). For instance, quantification and size determination showed great accuracy when used to identify exosomal secretion. Considering the advantages and disadvantages of all existing methods for EV isolation and identification, future analytical study is required to find promising techniques and methods that are all characterized by savings in terms of cost, availability, efficiency, labor, and time (Greening et al., 2015; Chen et al., 2019).

It is noteworthy that on comparing different cancer types, a significant diagnostic role of EVs was found in nervous system cancers. Our pooled results provide compelling evidence of a significant positive association between EVs and race. Our results also suggested promising accuracy for EV-mediated diagnosis in Europe compared with that in Asia and America, especially the patients aged more than 60 years. Considering the limitation of the small sample size of the American group, further large-scale studies among American populations should be designed to provide a comprehensive outcome.

We used the Duval and Tweedie's Trim and Fill model to analyses the possible publication bias. The key idea behind of this model is iterative procedure to remove the most extreme small studies from the positive side of the funnel plot, re-computing the effect size at each iteration until the funnel plot is symmetric about the (new) effect size. In theory, this will yield an unbiased estimate of the effect size. Too, major advantage of this approach is finding the best estimate of the unbiased effect size in intuitive visual display (Duval and Tweedie, 2000). Therefore, according incorporate Trim and Fill algorithm, we did not find any noticeable evidence for significant publication bias in our meta-analysis.

This study had some limitations. First, we only included articles published in English language, whereas published papers in other languages were ignored. Fundamentally, the meta-analysis results were based on unadjusted estimates because some studies did not provide detailed information to calculate the adjusted estimates. Definitely, chemotherapy and other therapeutic interventions can also alter the EV profile (Ab Razak et al., 2019; Wang et al., 2021), which has been out of control in reported studies. In this regard, further research should validate the diagnostic value of EV indices in cancer patients who receive different treatments, such as chemotherapy, immunotherapy, or radiotherapy, and differentiate between those treated with chemo radiation and those treated with monotherapy. Similarly, there are several reports showing that the clinical relevance of EVs is different between hematological malignancies and solid tumors (Iaccino et al., 2017; Maisano et al., 2020; Trino et al., 2021). In addition, the populations in the studies were not comprehensively represented, and there was a lack of research in the African populations. Researchers must seek to include a greater diversity of patient populations in future studies to analyze their EV profile. Furthermore, many confounding factors were not controlled for or reported in bias statistical results. Undoubtedly, well-designed large-scale studies with matched case-controls and functional studies are warranted in the future to validate these findings. Similarly, at a clinical level, suitable diagnostic examinations and appropriate randomized comprehensive experiments with different observational studies should be defined, to establish reliable diagnostic EV panel and guidelines for using EVs for early cancer detection.

## Conclusion

In conclusion, the data of the present meta-analysis show that high levels of EVs are reliable diagnostics biomarkers for the early detection of cancer. It has been determined that exosomal miRNA EVs are accurate diagnostic biomarkers in serum-based samples of patients with nervous system cancers.

## Data Availability Statement

The original contributions presented in the study are included in the article/Supplementary Material, further inquiries can be directed to the corresponding author.

## Author Contributions

S-yL, YL, and HH contributed to the data curation, conceptualization and methodology, review, and editing. SI and HH performed data validation, formal analysis, and editing. Q-lW gave the funding acquisition and validation and the final approval of the version to be submitted. SI is responsible for supervision, project administration, visualization, and paper writing—original draft. All authors contributed to the article and approved the submitted version.

## Funding

This work was supported by the Southwest Medical University (SWMU), Sichuan Province, China grants (No. 18080) that were awarded to SI.

## Conflict of Interest

The authors declare that the research was conducted in the absence of any commercial or financial relationships that could be construed as a potential conflict of interest.

## Publisher's Note

All claims expressed in this article are solely those of the authors and do not necessarily represent those of their affiliated organizations, or those of the publisher, the editors and the reviewers. Any product that may be evaluated in this article, or claim that may be made by its manufacturer, is not guaranteed or endorsed by the publisher.
