# Supplementary material for: Diagnostic Role of Extracellular Vesicles in Cancer: A Comprehensive Systematic Review and Meta-Analysis
Source: Front Cell Dev Biol. 2021 Oct 15;9:705791. doi: 10.3389/fcell.2021.705791 (PMC8555429; doi:10.3389/fcell.2021.705791)
Supplement: Supplementary file 1 [file Table_1.DOCX]

Supplementary Material

# Supplementary Table S1. Characteristic of extracellular vesicles in included studies.

| **First author (Ref.)** | **Type of Evs** | **EVs purification** | **EVs identification** | **Detection methods** | **Dysregulation** | **Diagnostic power** | | | | **EVs biomarkers** |
| --- | --- | --- | --- | --- | --- | --- | --- | --- | --- | --- |
|  |  |  |  |  |  | **TP** | **FP** | **FN** | **TN** |  |
| Que R.^a^ (Que et al., 2013) | miRNA | AUC^*^ | NA | qrt-PCR (TaqMan) | Up | 21 | 5 | 1 | 22 | miR-21 |
| Que R.^b^ (Que et al., 2013) | miRNA | AUC | NA | qrt-PCR (TaqMan) | Up | 16 | 2 | 6 | 25 | miR-17-5p |
| Cazzoli R. (Cazzoli et al., 2013) | miRNA | Exo Quick^**^ | NA | qrt-PCR (SYBR) | Up | 48 | 12 | 2 | 8 | miR-151a5p, miR-30a3p, miR200b-5p, miR-629, miR-100, miR-154-3p |
| Wang J.^a^ (Wang et al., 2014) | miRNA | AC | TEM | qrt-PCR (SYBR) | Up | 45 | 21 | 7 | 28 | miR-21 |
| Wang J.^b^ (Wang et al., 2014) | miRNA | AC | TEM | qrt-PCR (SYBR) | Up | 40 | 9 | 12 | 40 | HOTAIR |
| Madhavan B. (Madhavan et al., 2015) | miRNA | AC | FC | qrt-PCR (SYBR) | Up | 131 | 6 | 0 | 24 | miR-1246, miR-4644, miR-3976, miR-4306 |
| Ogata-Kawata H.^a^ (Ogata-Kawata et al., 2014) | miRNA | AUC | CD81^#^ | qrt-PCR (TaqMan) | Up | 59 | 2 | 29 | 9 | miR-let-7a |
| Ogata-Kawata H.^b^ (Ogata-Kawata et al., 2014) | miRNA | AUC | CD81 | qrt-PCR (TaqMan) | Up | 81 | 0 | 7 | 11 | miR-1299 |
| Ogata-Kawata H.^c^ (Ogata-Kawata et al., 2014) | miRNA | AUC | CD81 | qrt-PCR (TaqMan) | Up | 84 | 1 | 4 | 10 | miR-1246 |
| Ogata-Kawata H.^d^ (Ogata-Kawata et al., 2014) | miRNA | AUC | CD81 | qrt-PCR (TaqMan) | Up | 74 | 1 | 14 | 9 | miR-150 |
| Ogata-Kawata H.^e^ (Ogata-Kawata et al., 2014) | miRNA | AUC | CD81 | qrt-PCR (TaqMan) | Up | 54 | 1 | 34 | 10 | miR-21 |
| Ogata-Kawata H.^f^ (Ogata-Kawata et al., 2014) | miRNA | AUC | CD81 | qrt-PCR (TaqMan) | Up | 65 | 3 | 23 | 8 | miR-223 |
| Ogata-Kawata H.^g^ (Ogata-Kawata et al., 2014) | miRNA | AUC | CD81 | qrt-PCR (TaqMan) | Up | 81 | 0 | 7 | 11 | miR-23a |
| Matsumura T. (Matsumura et al., 2015) | miRNA | AUC | TEM | qrt-PCR (TaqMan) | Up | 133 | 2 | 76 | 14 | miR-19a |
| Melo S.^a^ (Melo et al., 2015) | Protein | AUC | TEM | qrt-PCR (SYBR) | Up | 19 | 41 | 59 | 23 | Glypican-1 |
| Melo S.^b^ (Melo et al., 2015) | Protein | AUC | TEM | qrt-PCR (SYBR) | Up | 143 | 38 | 62 | 47 | Glypican-1 |
| Butz H. (Butz et al., 2016) | miRNA | Isolation kit^***^ | NA | qrt-PCR (TaqMan) | Up | 86 | 15 | 23 | 36 | miR-126-3p, miR-449a |
| Chiam K.^a^ (Chiam et al., 2015) | miRNA | Exo Quick | NTA | qrt-PCR (TaqMan) | Up | 14 | 6 | 4 | 23 | miR-16-5p |
| Chiam K.^b^ (Chiam et al., 2015) | miRNA | Exo Quick | NTA | qrt-PCR (TaqMan) | Up | 17 | 0 | 1 | 29 | miR-16-5p, miR-253p, miR320a, let-7e-5p, miR15b-5p, miR-30a-5p, miR-3245p |
| Zhou X. (Zhou et al., 2017) | miRNA | Exo Quick | SRM | qrt-PCR (SYBR) | Up | 98 | 34 | 43 | 90 | miR-19b-3p, miR-21-5p, miR-221-3p, miR409-3p, miR-425-5p, miR-584-5p |
| Bryzgunova OE.^a^ (Bryzgunova et al., 2016) | miRNA | AC | TEM | qrt-PCR (SYBR) | Redistribution | 13 | 1 | 0 | 20 | miR-19b |
| Bryzgunova OE.^b^ (Bryzgunova et al., 2016) | miRNA | AC | TEM | qrt-PCR (TaqMan) | Redistribution | 11 | 3 | 1 | 19 | miR-16 |
| Samsonov R.^a^ (Samsonov et al., 2016) | miRNA | AUC | AFM | qrt-PCR (SYBR) | Up | 30 | 0 | 5 | 35 | miRNA-574-3p |
| Samsonov R.^b^ (Samsonov et al., 2016) | miRNA | AUC | AFM | qrt-PCR (SYBR) | Up | 23 | 2 | 12 | 33 | miR-141-5p |
| Samsonov R.^c^ (Samsonov et al., 2016) | miRNA | AUC | AFM | qrt-PCR (SYBR) | Up | 23 | 2 | 12 | 33 | miR-21-5p |
| Liu C. (Liu et al., 2016) | miRNA | Exo Quick | TEM | qrt-PCR (SYBR) | Down | 45 | 6 | 12 | 21 | miR-4772-3p |
| Zhang W.^a^ (Zhang et al., 2018) | miRNA | Isolation kit | FC | qrt-PCR (SYBR) | Up | 57 | 30 | 25 | 50 | miR-210 |
| Zhang W.^b^ (Zhang et al., 2018) | miRNA | Isolation kit | FC | qrt-PCR (SYBR) | Up | 66 | 19 | 16 | 61 | miR-1233 |
| Meng X.^a^ (Meng et al., 2016) | miRNA | AC | WB | qrt-PCR (TaqMan) | Up | 137 | 2 | 26 | 18 | miR-200a |
| Meng X.^b^ (Meng et al., 2016) | miRNA | AC | WB | qrt-PCR (TaqMan) | Up | 86 | 0 | 77 | 20 | miR-200b |
| Meng X.^c^ (Meng et al., 2016) | miRNA | AC | WB | qrt-PCR (TaqMan) | Up | 50 | 0 | 130 | 20 | miR-200c |
| Liu Q.^a^ (Liu et al., 2017) | miRNA | Exo Quick | TEM | qrt-PCR (SYBR) | Up | 81 | 2 | 115 | 19 | miR-21-5p |
| Liu Q.^b^ (Liu et al., 2017) | miRNA | Exo Quick | TEM | qrt-PCR (SYBR) | Up | 72 | 6 | 124 | 15 | miR-23b-3p |
| Liu Q.^c^ (Liu et al., 2017) | miRNA | Exo Quick | TEM | qrt-PCR (SYBR) | Up | 29 | 4 | 167 | 17 | miR-10b-5p |
| Machida T.^a^ (Machida et al., 2016) | miRNA | Isolation kit | NA | qrt-PCR (TaqMan) | Up | 8 | 0 | 4 | 13 | miR‑1246 |
| Machida T.^b^ (Machida et al., 2016) | miRNA | Isolation kit | NA | qrt-PCR (TaqMan) | Up | 9 | 3 | 3 | 10 | miR‑4644 |
| Sandfeld-Paulsen B. (Sandfeld-Paulsen et al., 2016) | Protein | AC | NA | NA | Up | 4 | 2 | 103 | 52 | Annexin A11 |
| Lea J. (Lea et al., 2017) | Protein | AC | FC | qrt-PCR (SYBR) | Up | 27 | 3 | 8 | 7 | CA-125 |
| Qu Z. (Qu et al., 2017) | miRNA | Exo Quick | TEM | qrt-PCR (SYBR) | Up | 17 | 3 | 13 | 7 | miR-665 |
| Lan F. (Lan et al., 2018) | miRNA | Exo Quick | NA | qrt-PCR (TaqMan) | Up | 52 | 3 | 8 | 40 | miR-301a |
| Wang N. (Wang et al., 2017) | miRNA | Exo Quick | TEM | qrt-PCR (TaqMan) | Down | 19 | 2 | 1 | 18 | miR-19b-3P,miR-106a-5p |
| Skotland T. (Skotland et al., 2017) | Protein | AC | NA | NA | Up | 9 | 5 | 6 | 8 | lipid species |
| Rodriguez M. (Rodriguez et al., 2017) | miRNA | AC | TEM | qrt-PCR (TaqMan) | Down | 28 | 0 | 0 | 19 | miR-196a-5p,miR-501-3p |
| Lai X. (Lai et al., 2017) | miRNA | AC | CD63 | qrt-PCR (TaqMan) | Up | 3 | 0 | 0 | 6 | miR-10b, miR-21, miR-30c, miR-181a |
| Jin X. (Jin et al., 2017) | miRNA | Isolation kit | CD63 | qrt-PCR (TaqMan) | Up | 38 | 1 | 9 | 12 | miR-let7b-5p, miR-let7e-5p, miR-23a3p |
| Yan S. (Yan et al., 2017) | miRNA | Isolation kit | NA | qrt-PCR (SYBR) | Up | 101 | 7 | 67 | 13 | miR-6803-5p |
| Shi M. (Shi et al., 2018) | miRNA | Isolation kit | NA | qrt-PCR (SYBR) | Down | 38 | 12 | 88 | 9 | miR-638 |
| Shiromizu T.^a^ (Shiromizu et al., 2017) | Protein | AUC | SRM | MS | Up | 88 | 2 | 18 | 52 | Annexin A3 |
| Shiromizu T.^b^ (Shiromizu et al., 2017) | Protein | AUC | SRM | MS | Up | 88 | 10 | 19 | 44 | Annexin A4 |
| Shiromizu T.^c^ (Shiromizu et al., 2017) | Protein | AUC | SRM | MS | Up | 92 | 2 | 15 | 52 | Annexin A11 |
| Shiromizu T.^d^ (Shiromizu et al., 2017) | Protein | AUC | SRM | MS | Up | 96 | 2 | 11 | 52 | Annexin A3 |
| Shiromizu T.^e^ (Shiromizu et al., 2017) | Protein | AUC | SRM | MS | Up | 4 | 10 | 103 | 44 | Annexin A4 |
| Arbelaiz A.^a^ (Arbelaiz et al., 2017) | Protein | AUC | TEM | MS | Up | 39 | 11 | 4 | 21 | AMPN |
| Arbelaiz A.^b^ (Arbelaiz et al., 2017) | Protein | AUC | TEM | MS | Up | 31 | 4 | 12 | 28 | VNN1 |
| Arbelaiz A.^c^ (Arbelaiz et al., 2017) | Protein | AUC | TEM | MS | Up | 36 | 9 | 7 | 23 | PIGR |
| Arbelaiz A.^d^ (Arbelaiz et al., 2017) | Protein | AUC | TEM | MS | Up | 28 | 9 | 1 | 23 | LG3BP |
| Arbelaiz A.^e^ (Arbelaiz et al., 2017) | Protein | AUC | TEM | MS | Up | 24 | 9 | 5 | 23 | PIGR |
| Tsukamoto M. (Tsukamoto et al., 2017) | miRNA | AUC | TEM | qrt-PCR (TaqMan) | Up | 150 | 9 | 176 | 21 | miR-21 |
| Xu Y. (Xu et al., 2017) | miRNA | AUC | TEM | qrt-PCR (SYBR) | Up | 47 | 23 | 13 | 38 | miR-145 |
| Yan S. (Yan et al., 2018a) | miRNA | Isolation kit | TEM | qrt-PCR (TaqMan) | Down | 64 | 16 | 128 | 23 | miR-638 |
| Goto T.^a^ (Goto et al., 2018) | miRNA | Exo Quick | CD63 | qrt-PCR (TaqMan) | Up | 26 | 4 | 6 | 18 | miR-191 |
| Goto T.^b^ (Goto et al., 2018) | miRNA | Exo Quick | CD63 | qrt-PCR (TaqMan) | Up | 21 | 3 | 11 | 19 | miR-21 |
| Goto T.^c^ (Goto et al., 2018) | miRNA | Exo Quick | CD63 | qrt-PCR (TaqMan) | Up | 23 | 3 | 9 | 19 | miR-451a |
| Pan C.^a^ (Pan et al., 2018) | miRNA | AC | CD63 | qrt-PCR (TaqMan) | Down | 65 | 5 | 41 | 24 | miR-21 |
| Pan C.^b^ (Pan et al., 2018) | miRNA | AC | CD64 | qrt-PCR (TaqMan) | Down | 66 | 8 | 40 | 21 | miR-100 |
| Pan C.^c^ (Pan et al., 2018) | miRNA | AC | CD65 | qrt-PCR (TaqMan) | Down | 68 | 4 | 38 | 25 | miR-200b |
| Pan C.^d^ (Pan et al., 2018) | miRNA | AC | CD66 | qrt-PCR (TaqMan) | Down | 59 | 9 | 47 | 20 | miR-320 |
| Wang X. (Wang et al., 2018) | miRNA | AC | TEM | qrt-PCR (SYBR) | Up | 37 | 6 | 8 | 24 | miR-210 |
| Yan S. (Yan et al., 2018b) | miRNA | Isolation kit | WB | qrt-PCR (SYBR) | Down | 102 | 24 | 40 | 26 | miR-6869-5p |
| Kanaoka R. (Kanaoka et al., 2018) | miRNA | AUC | TEM | qrt-PCR (TaqMan) | Up | 141 | 10 | 144 | 14 | miR-451a |
| Takahasi K. (Takahasi et al., 2018) | miRNA | AUC | TEM | qrt-PCR (TaqMan) | Up | 25 | 6 | 25 | 14 | miR-451a |
| Xu S. (Xu et al., 2019) | miRNA | AC | SRM | qrt-PCR (SYBR) | Down | 22 | 12 | 21 | 8 | miR-32 |
| Yu S.^a^ (Yu et al., 2019) | miRNA | Isolation kit | TEM | qrt-PCR (TaqMan) | Up | 10 | 8 | 2 | 24 | CDK6 |
| Yu S.^b^ (Yu et al., 2019) | miRNA | Isolation kit | TEM | qrt-PCR (TaqMan) | Up | 10 | 10 | 2 | 22 | RHOU |
| Sakaue T. (Sakaue et al., 2019) | Protein | AUC | TEM | MS | Up | 12 | 17 | 7 | 32 | CD133 |
| **Abbreviations:** EVs, extracellular vesicles; AUC, analytical ultracentrifuge; AC, ultracentrifuge; TEM, transmission electron microscope; AFM, atomic force microscopy; SRM, selected reaction monitoring; qrt-PCR, quantitative real-time reverse transcription PCR; MS, mass spectrometry; WB, western blot; Up, upregulation; Down, downregulation; NR, not reported; NA, not available and/or not measurable TP, true positive; FP, false positive; FN, false negative; TN, true negative;  * Using the differential ultracentrifugation range (>100,000 ×g (100,000–200,000 ×g) for 2 h) for isolation of different exosome isolation methods yield different amount of exosomes.(Patel et al., 2019)  ** Exo Quick is the optimized one-step solution for rapidly isolating extracellular vesicles from tissue culture media and urine for biomarker analysis  *** Isoaltion kit refer to the standard total extracellular vesicles isolation kit media and urine for biomarker analysis.  # CD81, CD63, CD64, CD65, and CD66 are a cell surface glycoprotein exosomal cell surface markers that is using mostly for isolation isolating exosomes from tissue culture media and urine for biomarker analysis.(Konoshenko et al., 2018) | | | | | | | | | | |

**REFERENCES**

Arbelaiz, A., Azkargorta, M., Krawczyk, M., Santos-Laso, A., Lapitz, A., Perugorria, M.J., et al. (2017). Serum extracellular vesicles contain protein biomarkers for primary sclerosing cholangitis and cholangiocarcinoma. *Hepatology* 66(4)**,** 1125-1143. doi: 10.1002/hep.29291.

Bryzgunova, O.E., Zaripov, M.M., Skvortsova, T.E., Lekchnov, E.A., Grigor'eva, A.E., Zaporozhchenko, I.A., et al. (2016). Comparative Study of Extracellular Vesicles from the Urine of Healthy Individuals and Prostate Cancer Patients. *PLoS One* 11(6)**,** e0157566. doi: 10.1371/journal.pone.0157566.

Butz, H., Nofech-Mozes, R., Ding, Q., Khella, H.W.Z., Szabo, P.M., Jewett, M., et al. (2016). Exosomal MicroRNAs Are Diagnostic Biomarkers and Can Mediate Cell-Cell Communication in Renal Cell Carcinoma. *Eur Urol Focus* 2(2)**,** 210-218. doi: 10.1016/j.euf.2015.11.006.

Cazzoli, R., Buttitta, F., Di Nicola, M., Malatesta, S., Marchetti, A., Rom, W.N., et al. (2013). microRNAs derived from circulating exosomes as noninvasive biomarkers for screening and diagnosing lung cancer. *J Thorac Oncol* 8(9)**,** 1156-1162. doi: 10.1097/JTO.0b013e318299ac32.

Chiam, K., Wang, T., Watson, D.I., Mayne, G.C., Irvine, T.S., Bright, T., et al. (2015). Circulating Serum Exosomal miRNAs As Potential Biomarkers for Esophageal Adenocarcinoma. *J Gastrointest Surg* 19(7)**,** 1208-1215. doi: 10.1007/s11605-015-2829-9.

Goto, T., Fujiya, M., Konishi, H., Sasajima, J., Fujibayashi, S., Hayashi, A., et al. (2018). An elevated expression of serum exosomal microRNA-191, - 21, -451a of pancreatic neoplasm is considered to be efficient diagnostic marker. *BMC Cancer* 18(1)**,** 116. doi: 10.1186/s12885-018-4006-5.

Jin, X., Chen, Y., Chen, H., Fei, S., Chen, D., Cai, X., et al. (2017). Evaluation of Tumor-Derived Exosomal miRNA as Potential Diagnostic Biomarkers for Early-Stage Non-Small Cell Lung Cancer Using Next-Generation Sequencing. *Clin Cancer Res* 23(17)**,** 5311-5319. doi: 10.1158/1078-0432.CCR-17-0577.

Kanaoka, R., Iinuma, H., Dejima, H., Sakai, T., Uehara, H., Matsutani, N., et al. (2018). Usefulness of Plasma Exosomal MicroRNA-451a as a Noninvasive Biomarker for Early Prediction of Recurrence and Prognosis of Non-Small Cell Lung Cancer. *Oncology* 94(5)**,** 311-323. doi: 10.1159/000487006.

Konoshenko, M.Y., Lekchnov, E.A., Vlassov, A.V., and Laktionov, P.P. (2018). Isolation of Extracellular Vesicles: General Methodologies and Latest Trends. *Biomed Res Int* 2018**,** 8545347. doi: 10.1155/2018/8545347.

Lai, X., Wang, M., McElyea, S.D., Sherman, S., House, M., and Korc, M. (2017). A microRNA signature in circulating exosomes is superior to exosomal glypican-1 levels for diagnosing pancreatic cancer. *Cancer Lett* 393**,** 86-93. doi: 10.1016/j.canlet.2017.02.019.

Lan, F., Qing, Q., Pan, Q., Hu, M., Yu, H., and Yue, X. (2018). Serum exosomal miR-301a as a potential diagnostic and prognostic biomarker for human glioma. *Cell Oncol (Dordr)* 41(1)**,** 25-33. doi: 10.1007/s13402-017-0355-3.

Lea, J., Sharma, R., Yang, F., Zhu, H., Ward, E.S., and Schroit, A.J. (2017). Detection of phosphatidylserine-positive exosomes as a diagnostic marker for ovarian malignancies: a proof of concept study. *Oncotarget* 8(9)**,** 14395-14407. doi: 10.18632/oncotarget.14795.

Liu, C., Eng, C., Shen, J., Lu, Y., Takata, Y., Mehdizadeh, A., et al. (2016). Serum exosomal miR-4772-3p is a predictor of tumor recurrence in stage II and III colon cancer. *Oncotarget* 7(46)**,** 76250-76260. doi: 10.18632/oncotarget.12841.

Liu, Q., Yu, Z., Yuan, S., Xie, W., Li, C., Hu, Z., et al. (2017). Circulating exosomal microRNAs as prognostic biomarkers for non-small-cell lung cancer. *Oncotarget* 8(8)**,** 13048-13058. doi: 10.18632/oncotarget.14369.

Machida, T., Tomofuji, T., Maruyama, T., Yoneda, T., Ekuni, D., Azuma, T., et al. (2016). miR1246 and miR4644 in salivary exosome as potential biomarkers for pancreatobiliary tract cancer. *Oncol Rep* 36(4)**,** 2375-2381. doi: 10.3892/or.2016.5021.

Madhavan, B., Yue, S., Galli, U., Rana, S., Gross, W., Muller, M., et al. (2015). Combined evaluation of a panel of protein and miRNA serum-exosome biomarkers for pancreatic cancer diagnosis increases sensitivity and specificity. *Int J Cancer* 136(11)**,** 2616-2627. doi: 10.1002/ijc.29324.

Matsumura, T., Sugimachi, K., Iinuma, H., Takahashi, Y., Kurashige, J., Sawada, G., et al. (2015). Exosomal microRNA in serum is a novel biomarker of recurrence in human colorectal cancer. *Br J Cancer* 113(2)**,** 275-281. doi: 10.1038/bjc.2015.201.

Melo, S.A., Luecke, L.B., Kahlert, C., Fernandez, A.F., Gammon, S.T., Kaye, J., et al. (2015). Glypican-1 identifies cancer exosomes and detects early pancreatic cancer. *Nature* 523(7559)**,** 177-182. doi: 10.1038/nature14581.

Meng, X., Muller, V., Milde-Langosch, K., Trillsch, F., Pantel, K., and Schwarzenbach, H. (2016). Diagnostic and prognostic relevance of circulating exosomal miR-373, miR-200a, miR-200b and miR-200c in patients with epithelial ovarian cancer. *Oncotarget* 7(13)**,** 16923-16935. doi: 10.18632/oncotarget.7850.

Ogata-Kawata, H., Izumiya, M., Kurioka, D., Honma, Y., Yamada, Y., Furuta, K., et al. (2014). Circulating exosomal microRNAs as biomarkers of colon cancer. *PLoS One* 9(4)**,** e92921. doi: 10.1371/journal.pone.0092921.

Pan, C., Stevic, I., Muller, V., Ni, Q., Oliveira-Ferrer, L., Pantel, K., et al. (2018). Exosomal microRNAs as tumor markers in epithelial ovarian cancer. *Mol Oncol* 12(11)**,** 1935-1948. doi: 10.1002/1878-0261.12371.

Patel, G.K., Khan, M.A., Zubair, H., Srivastava, S.K., Khushman, M., Singh, S., et al. (2019). Comparative analysis of exosome isolation methods using culture supernatant for optimum yield, purity and downstream applications. *Sci Rep* 9(1)**,** 5335. doi: 10.1038/s41598-019-41800-2.

Qu, Z., Wu, J., Wu, J., Ji, A., Qiang, G., Jiang, Y., et al. (2017). Exosomal miR-665 as a novel minimally invasive biomarker for hepatocellular carcinoma diagnosis and prognosis. *Oncotarget* 8(46)**,** 80666-80678. doi: 10.18632/oncotarget.20881.

Que, R., Ding, G., Chen, J., and Cao, L. (2013). Analysis of serum exosomal microRNAs and clinicopathologic features of patients with pancreatic adenocarcinoma. *World J Surg Oncol* 11**,** 219. doi: 10.1186/1477-7819-11-219.

Rodriguez, M., Bajo-Santos, C., Hessvik, N.P., Lorenz, S., Fromm, B., Berge, V., et al. (2017). Identification of non-invasive miRNAs biomarkers for prostate cancer by deep sequencing analysis of urinary exosomes. *Mol Cancer* 16(1)**,** 156. doi: 10.1186/s12943-017-0726-4.

Sakaue, T., Koga, H., Iwamoto, H., Nakamura, T., Ikezono, Y., Abe, M., et al. (2019). Glycosylation of ascites-derived exosomal CD133: a potential prognostic biomarker in patients with advanced pancreatic cancer. *Med Mol Morphol* 52(4)**,** 198-208. doi: 10.1007/s00795-019-00218-5.

Samsonov, R., Shtam, T., Burdakov, V., Glotov, A., Tsyrlina, E., Berstein, L., et al. (2016). Lectin-induced agglutination method of urinary exosomes isolation followed by mi-RNA analysis: Application for prostate cancer diagnostic. *Prostate* 76(1)**,** 68-79. doi: 10.1002/pros.23101.

Sandfeld-Paulsen, B., Aggerholm-Pedersen, N., Baek, R., Jakobsen, K.R., Meldgaard, P., Folkersen, B.H., et al. (2016). Exosomal proteins as prognostic biomarkers in non-small cell lung cancer. *Mol Oncol* 10(10)**,** 1595-1602. doi: 10.1016/j.molonc.2016.10.003.

Shi, M., Jiang, Y., Yang, L., Yan, S., Wang, Y.G., and Lu, X.J. (2018). Decreased levels of serum exosomal miR-638 predict poor prognosis in hepatocellular carcinoma. *J Cell Biochem* 119(6)**,** 4711-4716. doi: 10.1002/jcb.26650.

Shiromizu, T., Kume, H., Ishida, M., Adachi, J., Kano, M., Matsubara, H., et al. (2017). Quantitation of putative colorectal cancer biomarker candidates in serum extracellular vesicles by targeted proteomics. *Sci Rep* 7(1)**,** 12782. doi: 10.1038/s41598-017-13092-x.

Skotland, T., Ekroos, K., Kauhanen, D., Simolin, H., Seierstad, T., Berge, V., et al. (2017). Molecular lipid species in urinary exosomes as potential prostate cancer biomarkers. *Eur J Cancer* 70**,** 122-132. doi: 10.1016/j.ejca.2016.10.011.

Takahasi, K., Iinuma, H., Wada, K., Minezaki, S., Kawamura, S., Kainuma, M., et al. (2018). Usefulness of exosome-encapsulated microRNA-451a as a minimally invasive biomarker for prediction of recurrence and prognosis in pancreatic ductal adenocarcinoma. *J Hepatobiliary Pancreat Sci* 25(2)**,** 155-161. doi: 10.1002/jhbp.524.

Tsukamoto, M., Iinuma, H., Yagi, T., Matsuda, K., and Hashiguchi, Y. (2017). Circulating Exosomal MicroRNA-21 as a Biomarker in Each Tumor Stage of Colorectal Cancer. *Oncology* 92(6)**,** 360-370. doi: 10.1159/000463387.

Wang, J., Zhou, Y., Lu, J., Sun, Y., Xiao, H., Liu, M., et al. (2014). Combined detection of serum exosomal miR-21 and HOTAIR as diagnostic and prognostic biomarkers for laryngeal squamous cell carcinoma. *Med Oncol* 31(9)**,** 148. doi: 10.1007/s12032-014-0148-8.

Wang, N., Wang, L., Yang, Y., Gong, L., Xiao, B., and Liu, X. (2017). A serum exosomal microRNA panel as a potential biomarker test for gastric cancer. *Biochem Biophys Res Commun* 493(3)**,** 1322-1328. doi: 10.1016/j.bbrc.2017.10.003.

Wang, X., Wang, T., Chen, C., Wu, Z., Bai, P., Li, S., et al. (2018). Serum exosomal miR-210 as a potential biomarker for clear cell renal cell carcinoma. *J Cell Biochem*. doi: 10.1002/jcb.27347.

Xu, S., Li, J., Chen, L., Guo, L., Ye, M., Wu, Y., et al. (2019). Plasma miR-32 levels in non-small cell lung cancer patients receiving platinum-based chemotherapy can predict the effectiveness and prognosis of chemotherapy. *Medicine (Baltimore)* 98(42)**,** e17335. doi: 10.1097/MD.0000000000017335.

Xu, Y., Qin, S., An, T., Tang, Y., Huang, Y., and Zheng, L. (2017). MiR-145 detection in urinary extracellular vesicles increase diagnostic efficiency of prostate cancer based on hydrostatic filtration dialysis method. *Prostate* 77(10)**,** 1167-1175. doi: 10.1002/pros.23376.

Yan, S., Dang, G., Zhang, X., Jin, C., Qin, L., Wang, Y., et al. (2017). Downregulation of circulating exosomal miR-638 predicts poor prognosis in colon cancer patients. *Oncotarget* 8(42)**,** 72220-72226. doi: 10.18632/oncotarget.19689.

Yan, S., Jiang, Y., Liang, C., Cheng, M., Jin, C., Duan, Q., et al. (2018a). Exosomal miR-6803-5p as potential diagnostic and prognostic marker in colorectal cancer. *J Cell Biochem* 119(5)**,** 4113-4119. doi: 10.1002/jcb.26609.

Yan, S., Liu, G., Jin, C., Wang, Z., Duan, Q., Xu, J., et al. (2018b). MicroRNA-6869-5p acts as a tumor suppressor via targeting TLR4/NF-kappaB signaling pathway in colorectal cancer. *J Cell Physiol* 233(9)**,** 6660-6668. doi: 10.1002/jcp.26316.

Yu, S., Wang, X.S., Cao, K.C., Bao, X.J., and Yu, J. (2019). Identification of CDK6 and RHOU in Serum Exosome as Biomarkers for the Invasiveness of Non-functioning Pituitary Adenoma. *Chin Med Sci J* 34(3)**,** 168-176. doi: 10.24920/003585.

Zhang, W., Ni, M., Su, Y., Wang, H., Zhu, S., Zhao, A., et al. (2018). MicroRNAs in Serum Exosomes as Potential Biomarkers in Clear-cell Renal Cell Carcinoma. *Eur Urol Focus* 4(3)**,** 412-419. doi: 10.1016/j.euf.2016.09.007.

Zhou, X., Wen, W., Shan, X., Zhu, W., Xu, J., Guo, R., et al. (2017). A six-microRNA panel in plasma was identified as a potential biomarker for lung adenocarcinoma diagnosis. *Oncotarget* 8(4)**,** 6513-6525. doi: 10.18632/oncotarget.14311.
